# Case of Gunshot-Wound of Intestines Resection of Bowels—Death

**Published:** 1887-04

**Authors:** Geo. Cupples

**Affiliations:** San Antonio, Texas


DANIEL'S
### Texas Medical Journal.

PUBLISHED MONTHLY.—SUBSCRIPTION $2.00 A YEAR.

Vol. 2. AUSTIN, APRIL, 1887. No. 10.

### ORIGINAL ARTICLES.

CONTRIBUTED EXCLUSIVELY TO THIS JOURNAL.

*The Articles in this Department are accepted and published with the understanding that we are not responsible for, nor do we indorse the views and opinions of the writers by so doing.*

### CASE OF GUN SHOT WOUND OF INTESTINES—RESECTION OF BOWEL—DEATH.

*By Geo. Cupples, M. D., San Antonio.*

For Daniels Texas Medical Journal.

MRS. ——, October 21, shot herself with suicidal intent at 4 p. m. The bullet, 41 calibre, entered at the lower edge of the right hypochondrium, and was afterwards found to have traversed the peritoneal cavity, missing the liver, but grazing the interior or peritoneal surface of the abdominal parietes; it then penetrated the ileum, lodging under the integuments.

At 5:30 p. m. I was called upon by Dr. Lowry, who had first seen the patient, to operate. There was some collapse, apparently due to hemorrhage; pulse fairly good, but great alarm and apprehension.

With the assistance of Drs. Watts, Lowry, Kingsley, Barnitz and Johns, I made a laparotomy, expecting to find wounds of the intestine.

An incision of four inches was made in the linea alba, revealing large hemorrhage into the abdominal cavity. Very careful examination was made of the whole intestinal tract, which discovered two knuckles of ileum, penetrated by the bullet, causing four wounds, all within a length of bowel of about three inches.

The corresponding portion of mesentery having been secured by forceps, was excised, and a continuous suture made this absolutely secure. The portion of intestine of the same length, held in forceps, was separated by scissors, and the two ends having been carefully adjusted, the enterorrhaphy was completed by two rows of Lembert's suture, the whole comprising thirty-nine sutures. The hemorrhage ceased from the moment that the mesenteric supply was cut off from the wounded intestine.

The peritoneal cavity was thoroughly and carefully cleansed, and the abdominal wound closed by three silver wire, and four silk sutures, completing the operation in one hour and fifty-five minutes.

Her condition after the operation was not satisfactory; collapse, with vomiting ensued, and the patient died at 1 a. m., five hours from the completion of the operation.

